# Relationship between Methyl Tertiary Butyl Ether Exposure and Non-Alcoholic Fatty Liver Disease: A Cross-Sectional Study among Petrol Station Attendants in Southern China

**DOI:** 10.3390/ijerph13100946

**Published:** 2016-09-23

**Authors:** Jianping Yang, Qinzhi Wei, Xiaochun Peng, Xiaowu Peng, Jianhui Yuan, Dalin Hu

**Affiliations:** 1 Guangdong Provincial Key Laboratory of Tropical Disease Research, Department of Environmental Health, School of Public Health, Southern Medical University, Guangzhou 510515, China; doctoryangjp@163.com; 2 Department of Occupational Health, Baoan Center for Disease Control and Prevention of Shenzhen, Shenzhen 518100, China; 3 Guangdong Provincial Key Laboratory of Tropical Disease Research, Department of Toxicology, School of Public Health, Southern Medical University, Guangzhou 510515, China; cnwei99@163.com; 4 South China Institute of Environmental Sciences, Ministry of Environmental Protection, Guangzhou 510655, China; xchpeng@126.com (X.C.P.); pengxiaowu@scies.org (X.W.P.); 5 Department of Toxicology, Shenzhen Center for Disease Control and Prevention, Shenzhen 518055, China; jianhui_yuan@hotmail.com

**Keywords:** methyl tertiary butyl ether, environmental pollution, non-alcoholic fatty liver disease, epidemiology

## Abstract

Methyl tertiary butyl ether (MTBE)—A well known gasoline additive substituting for lead alkyls—causes lipid disorders and liver dysfunctions in animal models. However, whether MTBE exposure is a risk factor for non-alcoholic fatty liver disease (NAFLD) remains uncertain. We evaluate the possible relationship between MTBE exposure and the prevalence of NAFLD among 71 petrol station attendants in southern China. The personal exposure concentrations of MTBE were analyzed by Head Space Solid Phase Microextraction GC/MS. NAFLD was diagnosed by using abdominal ultrasonography according to the guidelines for the diagnosis and treatment of NAFLD suggested by the Chinese Hepatology Association. Demographic and clinical characteristics potentially associated with NAFLD were investigated. Mutivariate logistic regression analysis was applied to measure odds ratios and 95% confidence intervals (CI). The result showed that the total prevalence of NAFLD was 15.49% (11/71) among the study subjects. The average exposure concentrations of MTBE were 292.98 ± 154.90 μg/m^3^ and 286.64 ± 122.28 μg/m^3^ in NAFLD and non-NAFLD groups, respectively, and there was no statistically significant difference between them (*p* > 0.05). After adjusting for age, gender, physical exercise, body mass index (BMI), systolic blood pressure (SBP), diastolic blood pressure (DBP), alanine aminotransferase (ALT), white blood cell (WBC), total cholesterol (TC), triglycerides (TG), low-density lipoprotein (LDL), and high-density lipoprotein (HDL), the odds ratios were 1.31 (95% CI: 0.85–1.54; *p* > 0.05), 1.14 (95% CI: 0.81–1.32; *p* > 0.05), 1.52 (95% CI: 0.93–1.61; *p* > 0.05) in the groups (including men and women) with exposure concentrations of MTBE of 100–200 μg/m^3^, 200–300 μg/m^3^, and ≥300 μg/m^3^, respectively, as compared to the group (including men and women) ≤100 μg/m^3^. Our investigation indicates that exposure to MTBE does not seem to be a significant risk factor for the prevalence of NAFLD among petrol station attendants in southern China.

## 1. Introduction

Methyl tertiary butyl ether (MTBE)—A well known gasoline additive substituting for lead alkyls—is widely used in China and some other countries to increase the octane value of gasoline and reduce harmful emissions [1,2]. In recent years, with more and more people driving gasoline-fueled cars, the consumption of MTBE has dramatically increased, and due to its great volatility, it has become a ubiquitous environmental contaminant which has posed potential health risks to the population, especially the occupational workers with exposure to relatively high concentrations of MTBE [3]. For example, in petrol stations and their surrounding environments, the concentrations of MTBE may be thousands of times higher than in the general environment [3]. MTBE can be rapidly absorbed through respiratory and digestive systems, and rapidly distributed to all major tissues via the bloodstream [4]. Epidemiological investigation revealed that there was a significant positive correlation between ambient air concentrations and blood levels of MTBE [2,5]. The acute, subchronic, and chronic toxicities of MTBE have been explored, and the toxic effects of oxidative stress [6,7,8], DNA damage [5,6,7,8], DNA adducts [9,10], lipid disorder [11], abnormal liver functions [11], vascular lesions [12], and malignant tumors [13] induced by MTBE were found in animal tests. However, there is apparent uncertainty in the extrapolation of animal data to effects on humans [1,14,15]. The complaints of acute adverse health effects from populations exposed to MTBE include headache, cough, nausea, mucosal irritation, dizziness, etc. and these prompted scientific research on the toxicity of MTBE in many countries [3,16]. However, there was not sufficient evidence showing the reliable links between MTBE exposure and these complaints [17,18,19,20]. Because MTBE can cause the adverse effects of oxidative stress [6,7,8], lipid disorder [11], and abnormal liver functions [11] in animal models, we are interested in whether MTBE exposure is a risk factor for non-alcoholic fatty liver disease (NAFLD) in humans, which has not been reported.

NAFLD is the most typical chronic liver disease worldwide [21,22,23,24,25,26]. The spectrum of NAFLD ranges from simple hepatic steatosis to steatohepatitis, which may develop into cirrhosis and ultimately into liver carcinoma [22,23,24,25]. Identification of risk factors for the onset and development of NAFLD is very important for its prevention and control. Although metabolic disorder, body mass index (BMI), and waist circumference have been confirmed as major risk factors for NAFLD, the basic mechanism of hepatic steatosis remains unclear [22,23,25,27]. In recent decades, along with the acceleration of the process of urbanization and industrialization, the emissions of all kinds of environmental pollutants are dramatically increased. More and more evidence suggests that environmental pollutants such as diesel exhaust particles (DEP) [28], particulate matter (PM) suspended in the air [28,29], metals, and polychlorinated compounds [30,31,32], etc. are important risk factors for the progression of NAFLD. In the present study, we focus on the possible association between MTBE exposure and the prevalence of NAFLD in southern China.

## 2. Experimental Section 

### 2.1. Study Population and Ethical Permission

This study was carried out among petrol station attendants recruited from southern China during April to September 2014, all of whom had been working at the petrol stations for more than 3 years and had no history of chronic diseases and had not been taking any medicines for at least one year before this investigation. Some of the petrol station attendants (alcohol intake >0 g/day, hepatitis B antigen or hepatitis C virus antibody positive, autoimmune hepatitis, primary biliary cirrhosis, and other chronic liver disease with clear causes) were strictly excluded. A face-to-face investigation was carried out among the participants by trained interviewers with self-made questionnaires. The individual characteristic data, including age, gender, education, smoking habit, physical exercise, working types, and length of service, etc., were collected. A total of 71 eligible petrol station attendants were included in the final analysis. The procedures for the recruitment and data collection were approved by the local ethical research committee (No. 201158), and the purpose of the study was described in detail to each participant prior to obtaining written informed consent. 

### 2.2. Diagnosis of NAFLD

Abdominal ultrasonography is the most commonly used method for the diagnosis of NAFLD in epidemiological investigation in China [33,34,35]. According to the definition of NAFLD in the guidelines for the diagnosis and treatment of NAFLD suggested by the Chinese Hepatology Association, NAFLD was also diagnosed by using abdominal ultrasonography [36], and some populations (alcohol intake > 20 g/day, hepatitis B antigen or hepatitis C virus antibody positive, autoimmune hepatitis, primary biliary cirrhosis, and other chronic liver disease with clear causes) were strictly excluded. In order to control bias, diagnoses of NAFLD were performed by the same medical doctor with the same instrument in the Baoan Center for Disease Control and Prevention in the period between April to September 2014.

### 2.3. Basic Examinations and Laboratory Tests

The basic examination (including body mass index, systolic blood pressure, and diastolic blood pressure, etc.) for each subject was conducted with standard operating procedure in Baoan Center for Disease Control and Prevention from April to September, 2014. Eight milliliters of peripheral venous blood was collected from each selected subject at the end of work time by using a single-use syringe for laboratory tests (including alanine aminotransferase (ALT), white blood cell (WBC), platelet (PLT), blood urea nitrogen (BUN), serum creatinine (CREA), total glucose (GLU), serum globulins (GLO), serum albumin (ALB), hemoglobin (Hb), total cholesterol (TC), triglycerides (TG), low-density lipoprotein (LDL), and high-density lipoprotein (HDL), etc. which potentially associated with NAFLD). Each sample was labeled with serial number, date, and time of collection to avoid possible confusion.

### 2.4. Analysis of Personal Exposure Level of MTBE 

Personal exposure sampling of each participant was performed by using a charcoal-based organic vapor monitor (OVM; 3M 3520). MTBE was analyzed by using Head Space Solid Phase Microextraction GC/MS (model QP2010plus, Shimadzu, Japan), equipped with an AOC-20i+s automatic sampler (Shimadzu, Japan) and a Rtx-Wax column (30 m × 0.32 mm × 0.25 μm). The details were described previously [37].

### 2.5. Statistical Analysis

SPSS16.0 (SPSS Inc., IBM Company, Chicago, IL, USA) for Windows was used for statistical analysis. Descriptive analyses were conducted to characterize the demographics of the participants, including mean ± SD for continuous data and numbers and percentages for categorical data. The independent-sample Student’s *t*-test was adopted to assess between-group differences of quantitative materials. Chi-square test was conducted to compare categorical variables. Multivariate logistic regression analysis was conducted to estimate the odds ratio (OR) and 95% confidence interval (CI), in which the study population was classified into four groups according to the personal exposure concentrations of MTBE: ≤100 μg/m^3^, 100–200 μg/m^3^, 200–300 μg/m^3^, and ≥300 μg/m^3^, respectively. *p*-values were two-tailed, and the cutoff for statistical significance was set up at α = 0.05.

## 3. Results and Discussion

### 3.1. Demographic and Clinical Characteristics of Study Population 

As indicated in Table 1, a total of 71 eligible subjects were eventually recruited from different petrol stations in southern China, which consisted of 41 males (57.75%) and 30 females (42.25%). Among the study population, 11 were diagnosed with NAFLD. The prevalence of NAFLD was 24.39% (10/41) in males, 3.33% (1/30) in females, there was a statistically significant difference between them (*p* = 0.039), and the total prevalence of NAFLD was 15.49% (11/71). It was reported that NAFLD has been found worldwide, and it is recognized as the most common chronic liver disease in China and Western countries, with a prevalence ranging from 3%–46% in the general population [38,39,40,41,42]. However, for different countries or different studies, there existed some apparent difference in the prevalence of NAFLD—the reason for which has not yet been fully clarified. In our study, demographic and clinical characteristics potentially associated with NAFLD (e.g., age, gender, education, smoking habit, physical exercise, working types, and length of service, etc.) were also investigated during April to September, 2014. The result showed that there was a statistically significant difference in age (*p* = 0.000), gender (*p* = 0.037), physical exercise (*p* = 0.046), body mass index (BMI, *p* = 0.000), systolic blood pressure (SBP, *p* = 0.000 ), diastolic blood pressure (DBP, *p* = 0.000 ), ALT (*p* = 0.000), WBC (*p* = 0.000), TC (*p* = 0.000), TG (*p* = 0.002), LDL (*p* = 0.012), and HDL (*p* = 0.023) between the NAFLD and non-NAFLD groups, and there were no significant differences in education (*p* = 0.785) , smoking habit (*p* = 0.680), working types (*p* = 0.917), length of service (*p* = 0.365), PLT (*p* = 0.071), BUN (*p* = 0.067), CREA (*p* = 0.190), GLU (*p* = 0.076), GLO (*p* = 0.097), ALB (*p* = 0.061), and Hb (*p* = 0.053) between the two groups. Our study is consistent with the reported data [34,38,39,40,41,42]: for example, our study indicates that there was a higher prevalence of NAFLD in men (10/41 = 24.39%) than in women (1/30 = 3.33%) (*p* = 0.037), the reason for which may be the effect of estrogen on the amount and distribution of fat in men and women, and different blood lipid levels in men and women may directly affect the prevalence of NAFLD [34].

### 3.2. Personal Exposure Concentrations of MTBE among Petrol Station Attendants 

**Figure 1:**
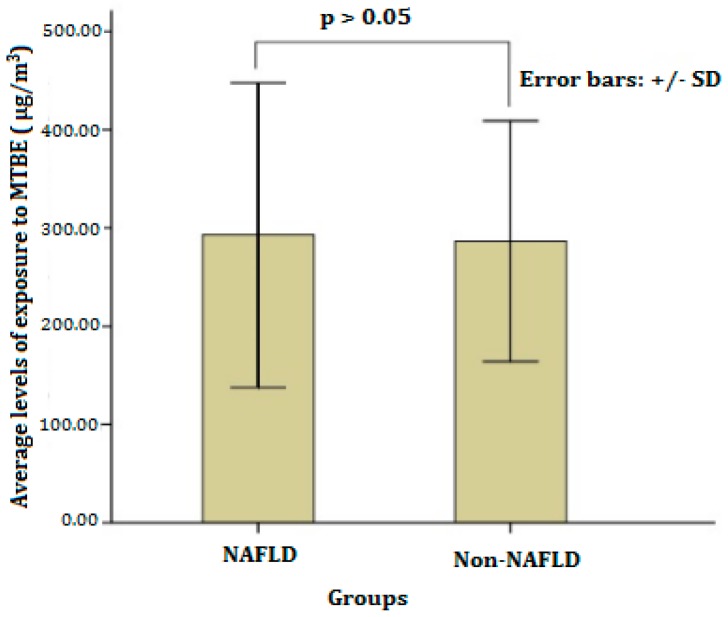
Average levels of exposure to methyl tertiary butyl ether (MTBE) in the NAFLD and Non-NAFLD groups.

Personal exposure concentrations of MTBE among petrol station attendants were analyzed by using Head Space Solid Phase Microextraction (HSSPME) GC–MS. The results showed that the average exposure concentrations of MTBE were 292.98 ± 154.90 μg/m^3^ and 286.64 ± 122.28 μg/m^3^ in NAFLD and non-NAFLD groups, respectively, and there was no statistically significant difference between the the them (*p* > 0.05, as shown in Figure 1). These occupational exposure concentrations of MTBE among petrol station attendants were much higher than that of general residents in the same area of the Pearl River Delta in Southern China, which ranged from 0 to 1.25 μg/m^3^ [43]. Fortunately, all of the observed values in each group did not exceed the ACGIH (American Conference of Governmental and Industrial Hygienists) Threshold Limit Value (TLV^®^) of MTBE (50 ppm; 1 ppm MTBE = 3.61 mg/m^3^; 50 ppm = 180.5 mg/m^3^ = 180,500 µg/m^3^). This situation may mainly be attributed to air purification equipment, which has been enforced to be established in each petrol station in southern China.

### 3.3. Multivariate-Adjusted Odds Ratios for the Prevalence of NAFLD in Groups with Different Exposure Concentrations of MTBE 

Multivariate logistic analysis showed that the crude odds ratios were 1.87 (95% CI: 0.91–2.11; *p* > 0.05), 1.51 (95% CI: 0.87–1.86; *p* > 0.05), and 1.91 (95% CI: 1.07–2.34; *p* < 0.05) in the groups (including men and women) with personal exposure concentrations of MTBE of 100–200 μg/m ^3^, 200–300 μg/m^3^, and ≥ 300 μg/m^3^, respectively, as compared to the group (including men and women) with the personal exposure concentration of MTBE ≤ 100 μg/m^3^. After adjusting for the confounding factors potentially associated with NAFLD (*p* < 0.05, as indicated in Table 1), including age, gender, physical exercise, BMI, SBP, DBP, ALT, WBC, TC, TG, LDL, and HDL, the odds ratios were 1.31 (95% CI: 0.85–1.54; *p* > 0.05), 1.14 (95% CI: 0.81–1.32; *p* > 0.05) and 1.52 (95% CI: 0.93–1.61; *p* > 0.05), respectively. At the same time, there was no significant association between MTBE exposure and the prevalence of NAFLD separately in men and women (*p* > 0.05, as shown in Table 2). Therefore, although the acute, subchronic, and chronic toxic effects of MTBE, such as lipid disorder [11], abnormal liver function [11], and oxidative stress [6,7,8], etc., were found in animal tests, there is no significant evidence showing an association between MTBE exposure and the prevalence of NAFLD in humans in our study. Of course, there were some limitations to this study, the first being that the exposure concentrations of MTBE among the subjects were relatively low (did not exceed the ACGIH TLV® of MTBE, 50 ppm); thus, it can hardly shed light on the association between relatively high exposure concentrations of MTBE and the prevalence of NAFLD. The second point is that our study is an epidemiological cross-sectional survey, which can not reveal the toxic effects related to long-term exposure to MTBE. The third point is that the sample size of our study is relatively small, which may limit the representativeness.

## 4. Conclusions 

Personal exposure concentrations of MTBE among petrol station attendants were relatively low, and do not seem to be a significant risk factor for the prevalence of NAFLD among petrol station attendants in southern China.

## Figures and Tables

**Table 1:** Analysis of factors potentially associated with non-alcoholic fatty liver disease (NAFLD).

Factors	NAFLD	Non–NAFLD	*X^2^/t* Value	*p* Value
Age (mean ± SD) (years)	39.75 ± 8.61	27.11 ± 6.97	8.760	0.000
Gender				
Male	10 (90.91%)	31 (51.67%)	4.369 (continuity correction)	0.037
Female	1 (9.09%)	29 (48.33%)		
Education				
High school	8 (72.72%)	49 (81.67%)	0.074 (continuity correction)	0.785
College	3 (27.28)	11 (18.33%)		
Smoking habit				
Yes	3 (27.27%)	10 (16.67%)	0.170 (continuity correction)	0.680
No	8 (72.73%)	50 (83.33%)		
Physical exercise				
Yes	1 (9.09%)	28 (46.67%)	3.988 (continuity correction)	0.046
No	10 (90.91%)	32 (53.33%)		
Working types				
Oil suppliers	9 (81.82%)	53 (88.33%)	0.011 (continuity correction)	0.917
Office workers	2 (18.18%)	7 (11.67%)		
Length of service (years)	5.60 ± 2.51	4.68 ± 2.03	0.936	0.365
BMI (kg/m^2^)	25.63 ± 1.46	21.29 ± 1.98	17.637	0.000
SBP (mmHg)	126.64 ± 10.32	109.45 ± 9.67	14.971	0.000
DBP (mmHg)	80.03 ± 7.34	71.25 ± 6.87	11.670	0.000
ALT (U/L)	36.54 ± 6.75	22.17 ± 5.71	9.584	0.000
WBC (×10^9^/L)	7.16 ± 2.64	5.69 ± 1.67	8.483	0.000
PLT (×10^9^/L)	220 ± 30.38	203 ± 23.77	21.371	0.071
BUN (mmol/L)	5.61 ± 1.92	4.63 ± 1.37	7.692	0.067
CREA (μmol/L)	87.61 ± 23.15	81.59 ± 21.64	13.593	0.190
GLU (mmol/L)	5.37 ± 1.62	5.13 ± 1.35	8.691	0.076
GLO (g/L)	28.34 ± 7.65	27.97 ± 6.8	16.452	0.097
ALB (g/L)	46.74 ± 8.15	44.69 ± 7.98	18.723	0.061
Hb (g/L)	150.79 ± 12.67	145.93 ± 11.77	17.871	0.053
TC (mmol/L)	5.26 ± 0.87	4.07 ± 0.73	7.572	0.000
TG (mmol/L)	2.17 ± 0.81	1.41 ± 0.30	5.033	0.002
LDL (mmol/L)	2.51 ± 0.71	2.11 ± 0.52	6.971	0.012
HDL (mmol/L)	1.21 ± 0.12	1.51 ± 0.22	−2.722	0.023

BMI: body mass index; SBP: systolic blood pressure; DBP: diastolic blood pressure; ALT: alanine aminotransferase; WBC: white blood cell; PLT: platelet; BUN: blood urea nitrogen; CREA: serum creatinine; GLU: total glucose; GLO: serum globulins; ALB: serum albumin; Hb: hemoglobin; TC: Total cholesterol; TG: triglycerides; LDL: low-density lipoprotein; HDL: high-density lipoprotein.

**Table 2:** Odds ratios for the prevalence of NAFLD among groups with different MTBE exposure concentrations.

MTBE (μg/m^3^)	Gender	n	Crude			Adjusted ^c^		
			OR ^a^	95% CI ^b^	*p* Value	OR	95% CI	*p* Value
≤100	M	4	1.00	1.00–1.00	-	1.00	1.00–1.00	-
	W	7	1.00	1.00–1.00	-	1.00	1.00–1.00	-
	T	11	1.00	1.00–1.00	-	1.00	1.00–1.00	-
100–200	M	4	1.93	0.78–2.31	>0.05	1.64	0.84–1.83	>0.05
	W	8	0.97	0.85–1.41	>0.05	1.17	0.79–1.32	>0.05
	T	12	1.87	0.91–2.11	>0.05	1.31	0.85–1.54	>0.05
200–300	M	25	1.68	0.78–1.89	>0.05	1.32	0.80–1.63	>0.05
	W	9	0.93	0.81–1.33	>0.05	1.02	0.79–1.26	>0.05
	T	34	1.51	0.87–1.86	>0.05	1.14	0.81–1.32	>0.05
≥300	M	7	1.35	0.77–2.41	>0.05	1.21	0.77–1.73	>0.05
	W	7	1.27	0.83–1.67	>0.05	1.11	0.75–1.41	>0.05
	T	14	1.91	1.07–2.34	<0.05	1.52	0.93–1.61	>0.05

M = Man; W = Woman; T = M + W; **^a^**: OR = Odds ratio; **^b^**: CI = Confidence interval; **^c^**: Adjusted for age, gender, physical exercise, BMI, SBP, DBP, ALT, WBC, TC, TG, LDL, and HDL, which potentially associated with NAFLD (*p* < 0.05, as indicated in Table 1).
